# The Sense of Sounds: Brain Responses to Phonotactic Frequency, Phonological Grammar and Lexical Meaning

**DOI:** 10.3389/fpsyg.2019.00681

**Published:** 2019-03-28

**Authors:** Susana Silva, Marina Vigário, Barbara Leone Fernandez, Rita Jerónimo, Kai Alter, Sónia Frota

**Affiliations:** ^1^Neurocognition and Language Group, Center for Psychology at University of Porto, Porto, Portugal; ^2^Center of Linguistics, School of Arts and Humanities, University of Lisbon, Lisbon, Portugal; ^3^Department of Psychology, Università Degli Studi di Torino, Turin, Italia; ^4^Instituto Universitário de Lisboa (ISCTE-IUL), CIS-IUL, Lisbon, Portugal; ^5^Institute of Neuroscience, Newcastle University, Newcastle upon Tyne, United Kingdom

**Keywords:** phonological processing, words, nonwords, pseudowords, phonotactic frequency, lexical-semantic processing, ERPs

## Abstract

Two outstanding questions in spoken-language comprehension concern (1) the interplay of phonological grammar (legal vs. illegal sound sequences), phonotactic frequency (high- vs. low-frequency sound sequences) and lexicality (words vs. other sound sequences) in a meaningful context, and (2) how the properties of phonological sequences determine their inclusion or exclusion from lexical-semantic processing. In the present study, we used a picture-sound priming paradigm to examine the ERP responses of adult listeners to grammatically illegal sound sequences, to grammatically legal sound sequences (pseudowords) with low- vs. high-frequency, and to real words that were either congruent or incongruent to the picture context. Results showed less negative N1-P2 responses for illegal sequences and low-frequency pseudowords (with differences in topography), but not high-frequency ones. Low-frequency pseudowords also showed an increased P3 component. However, just like illegal sequences, neither low- nor high-frequency pseudowords differed from congruent words in the N400. Thus, phonotactic frequency had an impact before, but not during lexical-semantic processing. Our results also suggest that phonological grammar, phonotactic frequency and lexicality may follow each other in this order during word processing.

## Introduction

Mapping sound into meaning, i.e., semantic processing, is the ultimate goal in spoken language comprehension. Knowledge on the time course and mechanisms of spoken language comprehension has greatly advanced in the last decades, mostly due to the temporal resolution of Event-Related-Potentials (ERP) methods and their ability to tap into covert processes (Kutas and Van Petten, 1994; Kutas et al., 2006). A well-known ERP signature of lexical-semantic processing is the N400 component (Kutas and Federmeier, 2011). The N400 shows increased amplitude when the integration of word meaning in a given context is more problematic, such as when a semantically incongruent word is placed within a sentence (e.g., “The pizza was too hot to *cry*” vs. “The pizza was too hot to *eat*”). The N400 for words may be experimentally elicited in sentence context, as in the previous example (e.g., Van den Brink and Hagoort, 2004; Laszlo and Federmeier, 2009; Brunellière and Soto-Faraco, 2015; Payne et al., 2015), or by priming techniques (e.g., Holcomb and Neville, 1990; Desroches et al., 2009; Robson et al., 2017; Wiese et al., 2017; Haebig et al., 2018). These techniques use a prime word or a prime picture followed by the target word, with prime-target pairs being either semantically congruent or incongruent. In the present study, we used ERPs to explore the time course of the processing of sound sequences and their integration with lexical meaning. A picture-word priming paradigm was applied, in which the prime picture was followed either by spoken real words or nonsense words. Real words were either semantically congruent (e.g., word ‘flower’ following the picture of a flower), or semantically incongruent to the picture context (e.g., word ‘flower’ following the picture of a ball). Nonsense words were sound sequences without meaning that were either phonotactically illegal (nonwords) or phonotactically legal (pseudowords). Given that they do not carry meaning, nonsense words were never consistent with the picture context. Moreover, pseudowords could consist of high frequency or low frequency sound sequences. By examining ERP responses to the sound-picture pairings, we investigated the interplay of phonological grammar (legal vs. illegal sound sequences), phonotactic frequency (high- vs. low-frequency sound sequences) and lexicality (words vs. other sound sequences) on word processing.

In their attempts to reach word meaning, language users must deal with several processing issues relating to the properties of phonological patterns. Not all sound sequences are words, and, therefore, not all sequences are equal candidates for meaning assignment. First, sound sequences may differ in *phonological grammaticality*, depending on whether they do or do not comply with the sound combination (phonotactic) rules of the language (Kager and Pater, 2012). For instance, in European Portuguese the [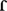] sound is not allowed to occur at the onset of words, even though it is legal at word medial or final positions. Thus, according to phonological grammar sound sequences are divided into legal (rule-compliant) and illegal ones, the latter sometimes named nonwords. Among legal sequences, there are pseudowords and words, which constitute different categories of *lexicality.* Pseudowords, like real words, comply with phonological grammar. However, they are not part of the lexicon of the language, in the sense that no shared meaning has been assigned to them. For instance, the sequence [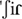] is possible in European Portuguese, but it does not appear in dictionaries because it has no meaning. Since both illegal sequences and pseudowords do not carry meaning, they are not a good match to a meaningful context. Finally, any legal phonological sequence, whether it is a word or a pseudoword, may be characterized by the frequency of its subsequences (sequences of phonemes) – this is phonotactic probability, also known as *phonotactic frequency*. For example, in European Portuguese the frequency of the sound combination [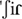] is lower than that of [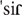], because [s] is frequently used in syllable initial and word initial position, unlike [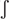] that occurs mostly in syllable final and word final position.

Although the formally-defined properties of phonological grammaticality, lexicality and phonotactic frequency are potential candidates to different processing stages, empirical findings are still insufficient to describe when and how each of these properties impacts the processing chain from sound to meaning. Two main questions remain unanswered. It is not known whether these properties show early effects on processing (before meaning is attained), and if so whether different properties yield different effects (ERP signatures). It is also unclear what properties may constrain semantic processing. In particular, it is unclear whether all sound sequences (words, pseudowords and nonwords) that do not match a picture prime are processed as incongruent (eliciting an N400), or whether some sound sequences, unlike words, are excluded from semantic processing. These questions persist because most ERP studies have considered phonological grammaticality, lexicality and phonotactic frequency separately, and none has looked at their joint influence on word processing. A few studies have addressed both phonological grammaticality and lexicality by combining illegal sequences, pseudowords and words – the latter congruent and incongruent with the semantic context (Friedrich and Friederici, 2005; Becker et al., 2014; Gansonre et al., 2018), but phonotactic frequency has been left out from the comparison. Studies focusing on phonotactic frequency, on the other hand, have shown that it impacts learning (Gonzalez-Gomez et al., 2013), word likeness judgments (Bailey and Hahn, 2001), pseudoword repetition (Vitevitch and Luce, 1998, 1999, 2005), ERPs (Bonte et al., 2005, 2007; Hunter, 2013; Wiese et al., 2017), and the dynamics of brain connectivity (Gow and Olson, 2015). Moreover, several studies have focused on the processing of real words only (e.g., Payne et al., 2015), or on legal versus illegal sound combinations (e.g., Domahs et al., 2009; Rossi et al., 2011), and a detailed account of the interplay of phonological grammaticality, lexicality and phonotactic frequency during the time course of word processing has not yet been provided.

Previous findings that speak to the first question highlighted above – the possible early effects of stimulus properties on word processing - suggest that illegal sequences and pseudowords elicit similar responses in the early ERPs, i.e., within the N1-P2 complex and before the time range of the N400 (Friedrich and Friederici, 2005; Moura et al., 2010). Consequently, phonological grammaticality seems to be irrelevant at these early stages. By contrast, early ERP components have responded to phonotactic frequency. For instance, Bonte et al. (2005, 2007) found an increased MMN to low-frequency sequences, while Hunter (2013) found an enlarged P200. Given that these were two separate lines of studies, and since pseudowords may consist of high or low-frequency sound sequences, we still know little on how phonotactic frequency may modulate the early processing of pseudowords and their association to illegal sequences.

Regarding the second question, namely what properties may constrain semantic processing, previous findings have mostly pointed to the inclusion of pseudowords in semantic processing (Kutas and Van Petten, 1994). Indeed, pseudowords have been reported to show an increased N400, similar to incongruent words, an unlike illegal sequences that show an N400 equivalent to that for congruent words (Bentin, 1987; Holcomb, 1993; Friedrich and Friederici, 2005; Kutas and Federmeier, 2011; Rossi et al., 2011; MacGregor et al., 2012; Kimppa et al., 2015; Gansonre et al., 2018). According to these findings, only illegal sequences are excluded from semantic processing. A few studies, however, have reported no differences between pseudowords and illegal sequences (Domahs et al., 2009; Laszlo and Federmeier, 2009), suggesting that both phonological grammar and lexicality constrain the processing of word meaning. N400 amplitude has also been shown to be sensitive to frequency effects, with lower frequency words eliciting larger N400s (Kutas and Federmeier, 2011; Winsler et al., 2018). So, phonotactic frequency is another factor that may modulate semantic processing. Again, we know little about the interplay between phonological grammar, lexicality and phonotactic frequency at this stage.

The present ERP study focused on the interplay of phonological grammar, phonotactic frequency and lexicality during the time course of word processing in a meaningful context created by means of a picture-word priming paradigm. This paradigm in particular was chosen to allow the replication of our study with infant and toddler populations and render the two sets of findings (adults and young children) comparable. By investigating how the properties of sound sequences influence the time course of word processing and determine lexical-semantic processing, we sought to answer two questions. Whether illegal sequences and pseudowords are equivalent in early processing stages, and whether an association between illegal sequences and pseudowords in early processing is modulated by the phonotactic frequency of pseudowords. To address this question, we examined early ERPs preceding the N400 time window. The second question was whether the inclusion of pseudowords in lexical-semantic processing is modulated by their phonotactic frequency, or whether pseudowords are instead included, or discarded, irrespective of phonotactic frequency. To address this question, we focused on the N400 time window.

## Materials and Methods

### Participants

Twenty-four healthy participants (16 women, age range: 18–34 years) voluntarily participated in the study in exchange for a small gift. They were all native European Portuguese (EP) speakers, right-handed, with normal or corrected-to-normal vision. They had no history of neurological, psychiatric, cognitive or language impairment, and none was taking drugs.

The study was carried out in accordance with the recommendations of the European Union Agency for fundamental Rights and the Declaration of Helsinki, with informed consent from all participants following Portuguese regulations. As part of the EBELa project (EXCL/MHC-LIN/0688/2012), the study was approved by the Comissão de Ética para a Saúde do Centro Hospitalar Lisboa Norte (Ref.^a^ DIRCLN-16JUL2014-208, Av. Professor Egas Moniz, 1649-035 Lisboa), and by the Comissão de Ética para a Saúde da Administração Regional de Saúde de Lisboa e Vale do Tejo (ARSLVT, Proc.015/CES/INV/2014, Av. Estados Unidos da América, 1749-096 Lisboa), Portugal.

### Stimuli

Visual stimuli were 22 pictures of objects familiar to young children, corresponding to words taken from the European Portuguese MacArthur-Bates Communicative Development Inventories (CDI) Short Forms (Frota et al., 2016, see Appendix 1). All words were thus commonly used words in the language. Auditory stimuli consisted of article-target sound sequence combinations (*uma flor*, ‘a flower’), produced by a female native speaker of European Portuguese. The stimuli were digitally recorded in a sound-attenuating chamber at 22050 Hz sampling frequency and bit depth of 16. Segmentation of audio files was manually performed with Praat (Boersma and Weenink, 2012) so that individual sound files began and ended with a silence of 100 ms.

Target sound sequences had either a monosyllabic or disyllabic shape, and included the 22 CDI words, plus 22 high-frequency pseudowords, 22 low-frequency pseudowords, and 22 illegal sequences (Appendix 1). Illegal sequences (IS) had an illegal prosodic word onset in EP, namely / 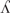, 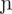, 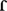 / (Vigário, 2003). Pseudowords consisted of legal sound combinations, and were thus possible word-like sequences. High-frequency pseudowords (HFPS) included consonants among the most frequent consonants used in syllable onset position ( / t, d, k, s, m, p, n /, with frequencies between 15 and 6%), whereas low-frequency pseudowords (LFPS) displayed the less frequent consonant onsets in the language ( / f, b, z, g, R, 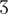, 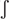, 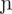, 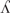 /, with frequencies below 3%), as computed from the FrePoP database (Frota et al., 2010). Finally, depending on the picture context, words were either congruent (CW), as in the case of cão [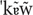] ‘dog’ following the picture of a dog, or incongruent (IW), as in the case of cão following the picture of a hat. Words (congruent and incongruent), pseudowords (high and low-frequency) and illegal sequences were matched for number of syllables: cão [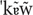] ‘dog’, [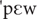] (HFPS), [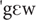] (LFPS), [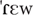] (IS); gato ['gatu] ‘cat’, ['nipu] (HFPS), ['Rafu] (LFPS), [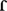] (IS). There were thus five conditions overall: IS, HFPS, LFPS, CW, IW. The properties of the stimuli by condition are summarized in Table 1.

**Table 1:** Properties of stimuli by condition, with examples.

**Phonological grammaticality**	Illegal	Legal	
	IS	LFPS, HFPS	CW, IW
**Phonotactic frequency**		Low High	
		LFPS, HFPS	
**Lexicality**		No meaning	Meaning
		LFPS, HFPS	CW, IW
**Example**	[ 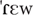 ]	[ 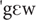 ] [ 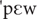 ]	[ 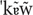 ] ‘dog’


*CW (Congruent Word), IW (Incongruent Word), HFPS (High-Frequency Pseudoword), LFPS (Low-Frequency Pseudoword), IS (Illegal Sequence).*

Each of the 22 pictures was paired with each of the target sound sequences producing the five experimental conditions (22 × 5). Since each picture-sound pair appeared twice, we had 220 trials in total (22 × 5 × 2). When pairing pictures with incongruent words, pseudowords and illegal sequences, picture names and sound stimuli never had the same onset sound (for example, the picture of a cat - ['gatu] in Portuguese - never appeared with a target sound sequence beginning with a [g] sound).

### Experimental Procedure and EEG Recordings

**FIGURE 1:**
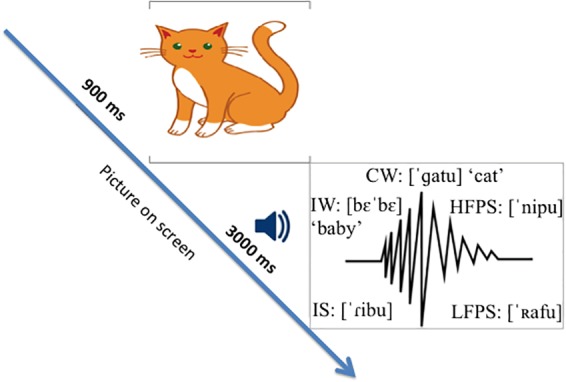
Picture-word priming paradigm used in the study.

The experiment was conducted in a sound-attenuating booth. Visual stimuli were presented in a 22-inch computer screen. Audio stimuli were presented through a loudspeaker at a constant and comfortable hearing level. Each trial consisted of a picture-sound pair presentation. The picture was presented 900 ms before the sound started and remained on screen for another 3000 ms during sound stimulus presentation until the end of the trial (Figure 1). The inter-trial-interval was 2000 ms. Picture-sound pairs were delivered in pseudo-randomized order using E-Prime software^1^. Participants were instructed to look at the pictures and hear the sounds. In order to minimize EEG artifacts, they were asked to blink when the picture stimulus disappeared, and to avoid body movements. The experiment lasted around 22 min.

The electroencephalogram (EEG) was continuously recorded with a Neuroscan system (SynAmps1, Compumedics Neuroscan, Abbotsford, VIC, Australia), from 29 active Ag/AgCl electrodes mounted on an elastic cap (Easycap 32 channels, Falk Minow, Herrching-Breitbrunn, Germany) according to the 10–20 system of electrode placement: FP1, FP2, F7, F3, Fz, F4, F8, FT7, FT3, FTz, FT4, FT8, T7, CT3, CTz, CT4, T8, TP7, CP3, CPz, CP4, TP8, P7, P3, Pz, P4, P8, O1, O2. The EEG recording was referenced on-line to the right mastoid, and re-referenced off-line to an average of the left and right mastoids. Impedances were kept below 5 KΩ, and the EEG signal was sampled at a rate of 500 Hz.

### EEG Preprocessing

EEG data were analyzed with the fieldtrip toolbox (Oostenveld et al., 2011) for Matlab^2^. Epochs were marked 200 ms before and 1000 ms after trigger points. Trials with ocular, muscle or movement artifacts were excluded from the analysis. Final trials were Notch filtered (50 Hz), band-pass filtered (0.01–40 Hz) and detrended. Baseline correction was applied from -200 ms to 0. Subject-level averages were obtained for the different conditions and channels, and these were later grand averaged.

### Statistical Analysis

A bottom-up analysis of Condition effects was performed, using Cluster Randomization Analysis (CRA) - a non-parametrical statistical test implemented in Fieldtrip (Maris and Oostenveld, 2007) - to define time windows with significant differences across the five conditions. A cluster is a group of adjacent channels showing significant differences between conditions along adjacent time samples. Each cluster is assigned a summed *T* value (sum-*T*, hereafter), corresponding to the sum of the *T* values obtained for each pair of samples. The sum-*T* values of each cluster were compared with a randomized null distribution of sum-*T* values, obtained from 4000 permutations. Clusters were accepted as significant when the actual sum-T was in the upper 5% tail of the randomized null distribution (critical Monte carlo *p*-value of 0.05).

Using the time windows provided by CRA, we then analyzed the averaged voltage values for each, grouping channels into six Regions Of Interest (ROIs), based on visual inspection: left anterior (LA), left central (LC), left posterior (LP), right anterior (RA), right central (RC), right posterior (RP). Repeated-measures ANOVAs were run with *Condition* (five levels: CW, IW, HFPS, LFPS, IS), *Laterality* (two levels: Left, Right) and *Caudality* (three levels: Anterior, Central, Posterior) as within-subject factors, using SPSS. In all ANOVAs, Greenhouse-Geisser corrections (Greenhouse and Geisser, 1959) were applied whenever necessary. Here, uncorrected degrees of freedom are reported.

For significant main effects of Condition without further interactions, we compared Condition levels (10 comparisons) with Bonferroni corrections, as embedded in the pairwise comparison method of SPSS. Effects of Caudality and Laterality were reported only when they interacted with the experimental manipulations (Condition). In this case, we first broke down the ANOVA into topographical levels to check for local Condition effects. When they existed, we compared the five Condition levels within each topographical level (anterior, central, posterior, or left, right) using paired-sample *t*-tests. Again, we applied Bonferroni corrections for 10 comparisons (significance level = significance level × 10, with a critical value of 0.05). We always report the corrected *p*-values.

## Results

**FIGURE 2:**
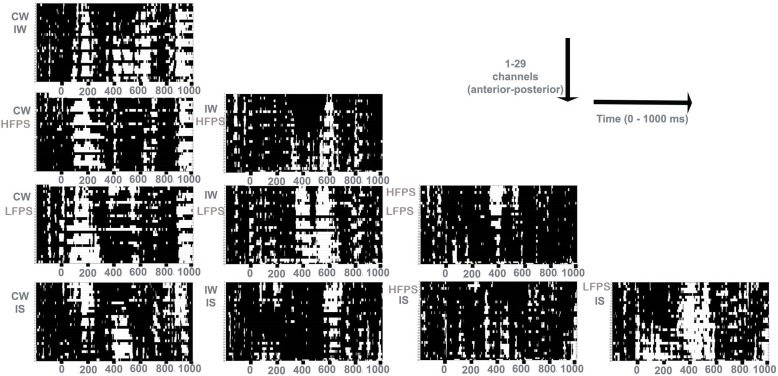
Probability associated with *t*-tests for paired comparisons, considering two conditions at a time (CW, Congruent Word; IW, Incongruent Word; HFPS, High-Frequency Pseudoword; LFPS, Low-Frequency Pseudoword; IS, Illegal Sequence). The rows of each panel represent channels, and the columns time bins. White cells represent significant differences.

The probabilities associated with all (simple) *T* values for each pair of samples – the basis for cluster computation in Cluster Randomization Analysis (CRA) - are shown in Figure 2 (non-significant in black; significant in white). The final results of CRA pointed to significant clusters defining four time windows (TWs) for analysis: 140–250 ms (TW I); 330–450 ms (TW II); 600–650 ms (TW III); 870–1000 ms (TW IV). We used these time windows in the following analyses.

### Decreased Negativity for IS (Anterior) and LFPS (Posterior) at TW I (140–250 ms)

**FIGURE 3:**
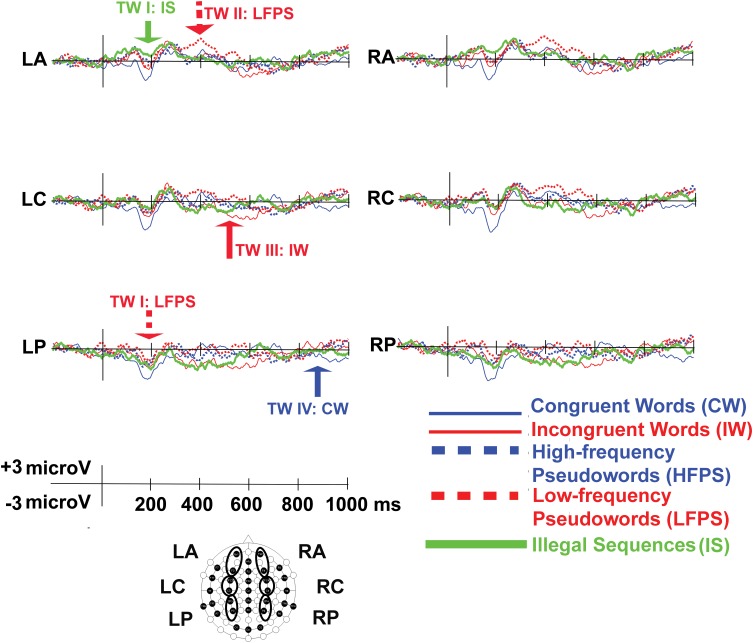
ERP waveforms for the six regions of interest (LA, left anterior; LC, left center; LP, left posterior; RA, right anterior; RC, right center; RP, right posterior). In time window (TW) I, IS and LFPS showed decreased negativity; In TW 2 only LFPS showed increased positivity; In TW III IW stood out for increased negativity; In TW IV, the same went for CW.

**FIGURE 4:**
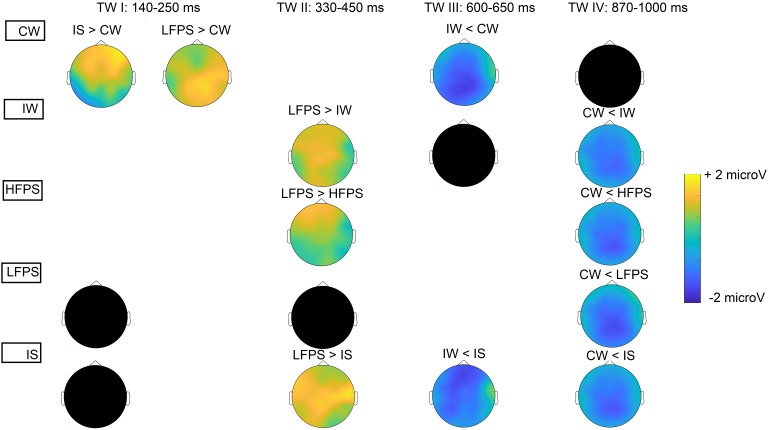
Topographic difference maps (significant differences across conditions). Columns indicate the four time windows. In each column, black circles represent the condition(s) that showed significantly increased/decreased potentials compared to others. Condition-level topographic plots are provided in Appendix 2.

The main effect of Condition was significant [*F*(4,92) = 3.65, *p* = 0.008, η^2^*p* = 0.14], and so was the Condition × Caudality interaction [*F*(8,184) = 3.29, *p* = 0.014, η^2^*p* = 0.13]. After Bonferroni corrections, anterior electrodes [Condition effect: *F*(4,92) = 3.36, *p* = 0.013, η^2^*p* = 0.13] showed significantly decreased negativity for IS compared to CW [*t*(23) = 2.22, *p* = 0.02, *d* = 0.86] and the same holds for central sites [Condition effect: *F*(4,92) = 3.62, *p* = 0.009, η^2^*p* = 0.14; IS vs. CW: *t*(23) = 3.29, *p* = 0.03, *d* = 0.76, Figure 3, 4, for condition-level topographic plots see Appendix 2]. At posterior sites, the main effect of Condition [*F*(4,92) = 3.83, *p* = 0.006, η^2^*p* = 0.14] related to decreased negativity for LFPS (instead of IS) compared to CW [*t*(23) = 3.68, *p* = 0.01, *d* = 0.89, Figure 3, 4].

### Increased Positivity for LFPS at TW II (330–450 ms)

There was a significant main effect of Condition [*F*(4,92) = 3.39, *p* = 0.012, η^2^*p* = 0.13], as well as an interaction between Condition and Caudality [*F*(8,184) = 2.64, *p* = 0.040, η^2^*p* = 0.12]. All Caudality levels showed Condition effects [anterior: *F*(4,92) = 2.70, *p* = 0.036, η^2^*p* = 0.10; central: *F*(4,92) = 3.43, *p* = 0.012, η^2^*p* = 0.13; posterior: *F*(4,92) = 3.73, *p* = 0.007, η^2^*p* = 0.14]. Increased positivity for LFPS was dominant (Figure 3, 4). This positivity was significantly larger than for HFPS at anterior sites [*t*(23) = 4.30, *p* < 0.001, *d* = 0.88] and marginally larger at central ones [*t*(23) = 2.80, *p* = 0.10, *d* = 0.11]. Responses to LFPS were also more positive than those to IS in the central region [*t*(23) = 3.06, *p* = 0.05, *d* = 0.31], and more positive than IW at central [*t*(23) = 3.46, *p* = 0.02, *d* = 0.72] and posterior [*t*(23) = 3.35, *p* = 0.003, *d* = 0.64] electrodes.

### Increased Negativity for IW at TW III (600–650 ms)

There was a significant main effect of Condition [*F*(4,92) = 3.97, *p* = 0.005, η^2^*p* = 0.15), without topographical interactions (Condition × Caudality: *p* > 0.30; Condition × Laterality: *p* > 0.10). Condition IW showed increased negativity (Figure 3, 4) compared to CW (*p* = 0.026) and to IS (*p* = 0.027).

### Increased Negativity for CW at TW IV (870–1000 ms)

The main effect of Condition [*F*(4,92) = 9.38, *p* < 0.001, η^2^*p* = 0.29] and the Condition × Caudality interaction [*F*(8,184) = 3.15, *p* = 0.022, η^2^*p* = 0.12] were both significant. There was marked negativity for CW (Figure 3, 4). Anterior electrodes [Condition effect: *F*(4,92) = 4.52, *p* = 0.002, η^2^*p* = 0.17] showed significantly increased negativity for CW compared to IW [*t*(23) = -3.62, *p* = 0.01, *d* = -0.81], to HFPS [*t*(23) = -3.45, *p* = 0.02, *d* = -0.90], and to IS [*t*(23) = -3.26, *p* = 0.03, *d* = -0.84]. At central electrodes, CW was more negative compared to all other conditions [CW vs. IW: *t*(23) = -4.54, *p* < 0.001, *d* = -0.91; CW vs. HFPS: *t*(23) = -4.39, *p* < 0.001, *d* = -1.10; CW vs. LFPS: *t*(23) = -4.42, *p* < 0.001, *d* = -1.06; CW vs. IS: *t*(23) = -3.22, *p* = 0.04, *d* = -0.72]. At posterior sites, CW was significantly more negative than IW [*t*(23) = -4.98, *p* < 0.001, *d* = -0.96], HFPS [*t*(23) = -4.52, *p* < 0.001, *d* = -1.12], and LFPS [*t*(23) = -5.03, *p* < 0.001, *d* = -1.18].

## Discussion

This study used ERPs to investigate the time course of the processing of sound sequences and their integration with lexical meaning. We addressed two questions: (1) Whether illegal sequences and pseudowords are equivalent in early processing stages, and a possible association between illegal sequences and pseudowords in early processing is modulated by the phonotactic frequency of pseudowords; (2) Whether high- and low-frequency pseudowords are both included in (like real words) or excluded from (similar to illegal sequences) lexical-semantic processing. To that end, we used a picture-word priming paradigm and compared the ERP responses to illegal sequences, low-frequency pseudowords, high-frequency pseudowords, picture-incongruent and picture-congruent words.

First, we examined early ERPs to test if illegal sequences and pseudowords are processed similarly, regardless of the phonotactic frequency of pseudowords. This was not the case: low-frequency pseudowords showed a response profile similar to illegal sequences in the earliest time window (140–250 ms: both diverged from congruent words), while high-frequency pseudowords did not. The decreased negativity pattern for illegal sequences and low-frequency pseudowords likely reflects the processing of unusual acoustic or phonetic patterns, a property that both illegal sequences and low-frequency pseudowords share, which is signaled by the N1-P2 complex (Näätänen and Picton, 1987; Desroches et al., 2009). In addition, the lower negativity displayed a different topography for low-frequency pseudowords (posterior) and illegal sequences (anterior). We suggest that the difference may reflect the processing of uncommon sound patterns that are well-formed (low-frequency pseudowords) and ill-formed (illegal sequences), thus signaling the early detection of phonologically illegal sound combinations.

Between 330 and 450 ms, a dissociation was found between low-frequency pseudowords and illegal sequences. Only low-frequency pseudowords showed increased (anterior) positivity, indicating the presence of a component from the P3 family (Polich, 2007), that marks an unexpected event. The fact that P3 was absent or decreased in illegal sequences suggests that illegal sequences may have been discarded from processing at this point, and listeners were dealing with unexpected *relevant* events. Both high-frequency pseudowords and words are phonologically relevant events too, but they are not unexpected or unfamiliar, and thus do not trigger increasing processing effort related to unfamiliar legal sound sequences. In this P3 time window, low- vs. high-frequency pseudowords were clearly dissociated.

Overall, our results add new elements to previous reports of early association between illegal sequences and pseudowords (Friedrich and Friederici, 2005; Moura et al., 2010). Although we found an association between low-frequency pseudowords and illegal sequences in the earliest time window, we also found that the ERP response had a different topography for low-frequency pseudowords and illegal sequences. In addition, an association was not found in the second time window (330–450 ms), where low-frequency pseudowords showed an increased P3-like component compared to illegal sequences. These findings suggest that phonological grammaticality (illegal sequences) and phonotactic frequency (low-frequency) show early effects on processing, but display different ERP signatures. Critically, the phonotactic frequency of pseudowords (low- vs. high-frequency) modulated the relation between pseudowords and illegal sequences.

Second, we examined how the properties of phonological sequences determine their inclusion or exclusion from lexical-semantic processing. In particular, we tested whether pseudowords are included or excluded, and if phonotactic frequency (high- vs. low-frequency pseudowords) may modulate lexical-semantic processing. Inclusion should be manifested as an increased late negativity (N400) compared to congruent words. We found no differences between high- and low-frequency pseudowords, and none of them differed from congruent words. In previous work contradictory findings had been reported, with most studies pointing to the inclusion of pseudowords (Bentin, 1987; Holcomb, 1993; Kutas and Van Petten, 1994; Friedrich and Friederici, 2005; Kutas and Federmeier, 2011; Rossi et al., 2011; MacGregor et al., 2012; Kimppa et al., 2015; Gansonre et al., 2018), and some studies reporting no differences between pseudowords and illegal sequences (Domahs et al., 2009; Laszlo and Federmeier, 2009). Although the current findings seem to indicate that pseudowords were excluded from semantic processing, in line with the latter set of studies, the fact is that pseudowords did not differ from incongruent words either, contrary to illegal sequences that differed from incongruent words but not from congruent ones. Therefore, while illegal sequences seem to have been clearly excluded from lexical-semantic processing, pseudowords in general showed an intermediate position. Importantly, the phonotactic frequency of pseudowords did not modulate semantic processing. These findings add to the current understanding of the effects of phonological grammaticality, phonotactic frequency and lexicality in the processing of sound sequences and their integration with meaning.

In the last time window, we found a negative response that was exclusive to congruent words. In the context of our paradigm, it likely reflects a wrap-up effect, indexing the internal verification of the match between congruent word and sound. Some N400 studies have found a similar index, which is known as the N800 (Nittono et al., 2002).

The overall picture concerning the effects of phonotactic frequency – at least when manipulated for pseudowords - is that it seems to have an impact on pre-semantic processing: It matters whether pseudowords consist of high- vs. low-frequency phoneme combinations, and pseudowords are thus not a homogenous category. However, when it comes to semantic processing, this influence seems to vanish.

Taken from a more global viewpoint, our results allow sketching a hypothetical time line characterizing how the effects of phonological properties unfold. First, both phonological grammar (i.e., the well-formedness of sound combinations) and phonotactic frequency (low- vs. high- frequency pseudowords) take the stage, likely defining what sound patterns are *uncommon* in the language (illegal sequences and low-frequency pseudowords are processed as uncommon patterns in the ERPs). Second, the truly impossible sound patterns (illegal sequences) are left out, and phonotactic frequency takes over: the *possible, though uncommon or unfamiliar patterns*, seem to be the focus now. Third and final, lexicality is the crucial factor to meaning. At this point, phonotactic frequency seems no longer to matter.

Our study raises a number of questions to be addressed in future research. One of these concerns the early dissociation of both illegal sequences and low-frequency pseudowords from congruent words, but not (always) from incongruent ones. Our interpretation for the early processing findings – that illegal sequences and low-frequency pseudowords represent unusual acoustic patterns – leaves unexplained the fact that these uncommon phonological sequences showed no consistent differences from incongruent words. One possibility is that the (early) negativity for congruent words was also partly due to the (also early) effect of facilitated lexical processing for primed (congruent) words (Friedrich and Friederici, 2005; Torkildsen et al., 2007). Disentangling these two effects – priming of congruent as negativity, unusual patterns as decreased negativity – is a challenge for future research.

Two other major challenges for the future concern the manipulation of phonotactic frequency within both pseudowords and words, together with further tests on the hypothetical time line of different phonological effects on word processing. These would be critical to confirm the present finding that listeners progressively switch their focus from establishing what sound patterns are well-formed to sensitiveness to unfamiliar (low frequency) vs. familiar (high-frequency) phonological patterns, and then from the familiarity of phonological patterns to the phonology of known words, that is, sound patterns with meaning. Last but not least, our findings may provide an adult reference for studies on the development of the processing of sound sequences in meaningful contexts, which take into account the interplay of phonological grammar, phonotactic frequency and lexical meaning.

## Data Availability

The datasets generated for this study are available on request to the corresponding author.

## Author Contributions

SF and MV conceived and designed the experiments. BF performed the experiments. RJ contributed to data collection. SS analyzed the data. SS, SF, and KA wrote the manuscript.

## Conflict of Interest Statement

The authors declare that the research was conducted in the absence of any commercial or financial relationships that could be construed as a potential conflict of interest.

## Figures and Tables

**Appendix 2:**
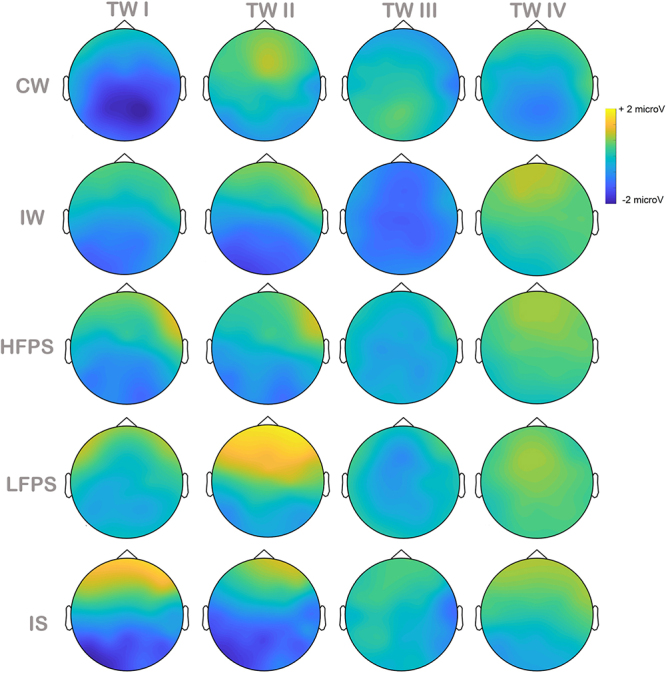
Topographic maps per time window and condition: CW (Congruent Word), IW (Incongruent Word), HFPS (High-Frequency Pseudoword), LFPS (Low-Frequency Pseudoword), IS (Illegal Sequence); TW I (140–250 ms), TW II (330–450 ms), TW III (600–650 ms), TW IV (870–1000 ms).

## Footnotes

1 https://pstnet.com/products/e-prime/

2 mathworks.com

## Appendix

**Appendix 1:** Auditory stimuli.
